# The use of pure and impure placebo interventions in primary care - a qualitative approach

**DOI:** 10.1186/1471-2296-12-11

**Published:** 2011-03-24

**Authors:** Rahel Fent, Thomas Rosemann, Margrit Fässler, Oliver Senn, Carola A Huber

**Affiliations:** 1 Institute of General Practice and Health Services Research, University of Zurich, Pestalozzistrasse 24, 8091 Zurich, Switzerland; 2 Institute of Biomedical Ethics, University of Zurich, Pestalozzistrasse 24, 8032 Zurich, Switzerland

## Abstract

**Background:**

Placebos play an important role in clinical trials and several surveys have shown that they are also common in daily practice. Previous research focused primarily on the frequency of placebo use in outpatient care. Our aim was to explore physicians' views on the use of placebos in daily practice, whereby distinction was made between pure placebos (substances with no pharmacological effect, e.g. sugar pills) and impure placebos (substances with pharmacological effect but not on the condition being treated, e.g. antibiotics in viral infections or vitamins).

**Methods:**

We performed semi-structured interviews with a sample of twelve primary care physicians (PCPs). The interview addressed individual definitions of a placebo, attitudes towards placebos and the participants' reasons for prescribing them. The interviews were transcribed and analysed using qualitative content analysis.

**Results:**

The definition of a placebo given by the majority of the PCPs in our study was one which actually only describes pure placebos. This definition, combined with the fact that most impure placebos were not regarded as placebos at all, means that most of the participating PCPs were not aware of the extent to which placebos are used in daily practice. The PCPs stated that they use placebos (both pure and impure) mainly in the case of non-severe diseases for which there was often no satisfactory somatic explanation. According to the PCPs, cases like this are often treated by complementary and alternative therapies and these, too, are associated with placebo effects. However, all PCPs felt that the ethical aspects of such treatment were unclear and they were unsure as to how to communicate the use of placebos to their patients. Most of them would appreciate ethical guidelines on how to deal with this issue.

**Conclusions:**

Many PCPs seem to be unaware that some of the drugs they prescribe are classified as impure placebos. Perceptions of effectiveness and doubts about the legal and ethical aspects of the use of placebos by PCPs may discourage their application. Dissemination of guidelines and consensus papers may be one approach, but it has to be acknowledged that the topic itself is in conflict with the PCPs' perception of themselves as professional and reliable physicians.

## Background

Placebos are commonly used in clinical trials and a number of surveys have shown that they are also common in daily practice [1]. The placebo effect has been examined in several studies [2-5]. Most physicians agree that the placebo effect plays a significant role, but that the use of placebos is often associated with uncertainty regarding the ethical dimensions of whether and how to communicate the use of a placebo to the patient.

Previous research focused mainly on quantitative aspects, such as the frequency of prescribing placebos in routine care. Questionnaire surveys from Denmark [6] and Israel [7] for instance showed that more than half of the physicians used placebos. In Denmark, 86% of the primary care physicians had prescribed a placebo at least once within the last year and about half of the physicians prescribe them on average once a month. However, the frequency of use in daily practice is only one important aspect of this issue - at least as important are the attitude, motivation and expectations of physicians.

In order to evaluate these qualitative aspects of placebo use, it is crucial to distinguish between pure placebos and impure placebos [8]. Pure placebos are substances or forms of treatment that have no pharmacological effect, e.g. sugar pills or saline infusions. Impure placebos have pharmacological effects, but the effect on the specific disease the substance is prescribed for has not been proven or is uncertain. Examples of impure placebos are vitamin infusions in the treatment of cancer or antibiotics for virally-caused common colds.

In the prescription of pure placebos there is a moral implication of "lying to the patient" and this could jeopardise the doctor-patient relationship. This aspect has frequently been criticised by ethicists. In contrast, the prescription of impure placebos seems easier to justify because interventions with these placebos may have some pharmacological or physical effect which impacts the patient's health in a positive manner [9].

Since all previous studies - with the exception of the qualitative study of Lynöe et al. from 1993 [10] - were carried out on the basis of surveys, little is known about the dilemmas, motives and uncertainties of the physicians or of the legal "grey zone" or expectations regarding the use of pure/impure placebos.

The aim of our study was to explore primary care physicians' attitudes, behaviour and experiences regarding the use of placebos in a qualitative approach. We explored whether, how and why they prescribe placebos in daily practice. Furthermore, we aimed to identify potentially significant distinctions that practising clinicians make between the prescription and legitimacy of pure vs. impure placebos.

## Methods

### Sampling

A non-randomised, convenience sample of practising PCPs from a region in Switzerland was used. PCPs were recruited during a symposium which did not focus on issues of placebo use. The symposium was organised by the Institute of General Practice and Health Services Research at the University of Zurich. A leaflet was distributed describing the aim of the study. Fourteen primary care physicians enlisted their names and addresses, most of them practising in or around Zurich. Of these participants, twelve were from rural practices and only two from urban ones.

### Interviews

We developed a semi-structured interview guide with open-ended questions that were categorised into six main domains (shown in Additional file 1).

Before starting the interviews, a pre-test was performed to ensure content validity of the interview guide. The interviews were conducted by RF during the period April - July 2009 at the physicians' own practice locations. No specific definition of the term "placebo" was given, since we intended to investigate how the physicians defined it themselves. Each interview lasted about 30 minutes and was recorded digitally. The interviewer ensured that every question was well understood and double-checked replies that were not entirely clear in order to avoid queries or misunderstandings later on. Where necessary, the interviewer asked additional questions to clarify the participant's opinion.

### Questions

Physicians were asked about their attitude, behaviour and experiences regarding the use of placebos as suggested in the moderator's guide. They were asked whether, how and why they use placebos in their daily practice, what they communicate to the patient, how they deal with the moral aspects of using placebos and whether they thought there was a need for recommendations or guidelines on the use thereof.

### Analysis

Without disclosing the identities of the participants, the interviews were transcribed literally and subsequently analysed according to the qualitative content analysis of Mayring [11]. ATLAS.ti-software [12] helped to code the interviews. Before starting the analysis, we established an initial categorising system based on the moderator's guide (shown in Table 1). During the process of analysis, categories were consistently modified and/or supplemented with (sub-) categories. In addition, a free category was created from the text and incorporated into the categorising system. With a view to heightening the objectivity of categorising and coding, the transcriptions were read and analysed by two coders (one clinician/researcher, one researcher) independently. The results were then compared. There were no notable discrepancies in the selection of relevant quotes or in coding. Minor differences in the two analyses were discussed between the coders and common agreement on coding was reached. After coding the interviews with ATLAS.ti, the program also allowed the content-analysis of the participants' opinions on the use of placebos. Quotes that were selected to be shown in the results section were translated in advance.

**Table 1:** Coding system

Themes and Categories	Subcategory (1)	Subcategory (2)
1. Physicians' definition of placebo and explanation of placebo effects		

2. Experiences with placebo use		

3. Placebo use in daily practice	Pure placebos	Impure placebos

4. CAM as placebo treatment	Phytotherapy	Homeopathy

5. Communication with the patient/Ethical issues		

6. Need for guidelines		

7. Further remarks		

### Ethics or Ethical approval

The Research Ethics Committee of the Canton of Zurich, Switzerland, approved the study. All information was treated confidentially.

## Results

Twelve of the fourteen enlisted primary care physicians finally agreed to participate. Our study sample consisted of eight general practitioners, two internists, one paediatrician and one psychiatrist. On the basis of the Swiss health care system, these practitioners can all be classified as primary care providers. The age of the participants ranged from 45 to 66 years with a mean age of 56 years, as shown in Table 2. Two out of twelve interviewed physicians were women.

**Table 2:** Details of the interviewed practitioners

Specialisation	Sex	Age
General medicine	Male	45

General medicine	Male	48

Internal medicine	Male	50

General medicine	Female	54

General medicine	Female	58

Internal medicine	Male	59

General medicine	Male	60

General medicine	Male	62

General medicine	Male	62

General medicine	Male	66

Paediatrician	Male	54

Psychiatry, Homeopathy	Male	56

### Theme 1: Definition of placebo

We asked physicians to state in their own words their definition of placebo and of the placebo effect, their attitude towards placebos and their own explanation of how they might work. When asked about how they would define the word "placebo", the majority of PCPs stated that a placebo is a dummy drug without substance. Some commented that a placebo does not necessarily need to be a pill, but can also be an injection or treatment. All but one of the interviewed PCPs discussed only pure placebos, and only one mentioned that there are different kinds of placebos, distinguishing pure from impure placebos, defining this as follows:

*'There are true placebos that have some random fantasy name and have nothing in them, and then there are impure placebos, for example vitamin compounds, that may have some effect, but one prescribes them intending to induce a placebo effect.' *(PCP 12, male, 59 years)

It can thus be deduced that the majority of PCPs are not sufficiently informed to distinguish between pure and impure placebos and that they are not aware of the (impure) placebo effect of vitamins or antibiotics.

In addition, physicians' statements clearly indicated an ambivalent attitude toward placebos. For example, one PCP said: *'I would say the effect is both positive and negative. I think that a GP consultation in itself often creates a placebo effect without the physician even being aware of it. Our authority, charisma or empathy are often a lot more effective than the medication we prescribe. The negative aspect, on the other hand, is that I feel as if I am deceiving the patient or not confronting him with the truth.' *(PCP 8, female, 58 years)

PCPs are very ambivalent in their evaluation of the placebo effect. On the one hand, they emphasised the supplemental benefit of the placebo effect in every treatment. On the other hand, they mentioned the negative aspect of placebo use. PCPs are aware of the fact that they have a great deal of latitude in providing placebo treatment without patient consent. Due to this lack of consent, the physicians often associated placebo use with deception of the patient and thus viewed the prescribing of placebos as problematic. They emphasised the negative aspect of feeling that they were lying to their patients.

### Theme 2: Experience with placebo effects

The category "experience" reflects physicians' experiences with the use of placebos and provides information as to whether they have ever observed a placebo effect in their daily practice. All PCPs stated that placebo effects do exist. Nevertheless, many PCPs deny ever having observed a placebo effect in their own daily practice. In the course of the interview, however, many of them reported several specific situations in which a placebo effect had occurred. The physicians were not aware of this effect. They underestimated the possible impact of placebos in their daily practice.

For example, one primary care physician reported cases where patients felt immediate relief from their complaint following an injection with lidocaine and cortisone, although pharmacologically, the medication cannot act that quickly.

*'So, for example, when a patient comes to me with torticollis, i.e. with strained neck muscles, and I give him an injection of lidocaine with a little cortisone, I have often observed that as soon as the injection has been administered and the patient has been escorted outside, he reports feeling that the injection has already taken effect and that his ability to move has improved. The same has also been observed with regard to lower back pain. Well, in my opinion, the drug cannot act so fast.' *(PCP 7, male, 45 years)

### Theme 3: Use of placebos among PCPs with placebo experience

"Placebo use" as a category included physicians' statements about the use of both actual placebos and of placebos in the form of complementary and alternative medicine (CAM) in their daily practice.

### Subtheme: Use of placebos in daily practice

PCPs expressed ambivalence towards using placebos in daily practice. On the one hand, most of them believed that placebos provide beneficial treatment. On the other hand, they were often concerned about what to tell the patient. Nearly all were afraid of the potential harm of deception. When asked whether there are specific situations (disease- or patient-specific characteristics) in which they use placebos, all physicians stated that the response to placebos varies according to clinical condition or gender. They use placebos most often when they believe the patient has a less serious or psychogenic disorder. PCPs named fractures or malignant tumours as examples in which they believed placebos would not work at all. They tend to use placebos in particular in situations where a strong psychological strain is assumed. Other reasons given were "to calm the patient down" and to avoid adverse drug reactions. Some of them stated that women respond better than men or children. One primary care physician stated that intelligence has no influence on the placebo effect.

Only two physicians affirmed having sugar pills in stock in order to use them as placebos. Two PCPs said that they use saline injections for patients with rheumatic complaints who request an injection against the pain. Other PCPs argued in favour of using placebos in order to avoid drug addiction to pain killers or sleeping pills. One PCP emphasised that he only uses pure placebos on patients he has known for a long time and who he judges to be suitable for such treatment. Furthermore, it was interesting that two PCPs used pure placebos in a diagnostic procedure to investigate the origin or the nature of the particular disease.

The physician's decision on whether to use an impure placebo is based, on the one hand, on his perception of the patient's expectation to be given "something" for his complaint and, on the other, his conviction that patients should take responsibility for their own health and be aware that not all complaints can be by taking a pill.

'*I prefer to administer placebos in liquid form. For example, if a patient comes to me with a mental problem and I do not yet consider psychiatric drugs to be necessary, I hand him a glass of water, telling him that it contains a substance that will calm him down. Sometimes I have observed that a patient will actually calm down a little.' *(PCP 4, male, 62 years)

*'People come and expect me to give them something. Although I know that what I am giving them won't really help, I feel I have to do something rather than nothing and what I give them certainly won't do them any harm' ' *(PCP 5, male, 54 years)

Some PCPs reported a strong need on the part of patients to be taken seriously. Patients expect the physician to administer a drug and not to simply do nothing.

### Subtheme: CAM as placebo treatment

Eleven of the twelve physicians stated that they use phytotherapy, homeopathy or both. Phytotherapy was considered to produce both pharmaceutical and substantial placebo effects. In contrast to this, eight PCPs stated that homeopathy works solely by placebo effect:

*'I think phytotherapy is also a form of pharmacological therapy, simply not with pure substances. There is classical homeopathy, where I think the patient's feeling of being cared for and the relationship to the physician account for much of the effect. And then there is also 'over-the-counter homeopathy' at the pharmacy, in which I think that the placebo effect is much more important*. (PCP 5, male, 54 years)

The effect of homeopathy was seen as a result of a good doctor-patient relationship built on respect and trust of the physician, thus improving health outcomes.

### Theme 4: Communication with the patient

We defined "communication with the patient" as the way in which the PCP communicates with the patient when he uses a placebo and how the PCP deals with the moral aspect of doing so, in particular with his perception of "lying" to the patient. The majority of the PCPs in the study only gave their patients vague and very general information when they used placebos. They claimed to be in a conflict. On the one hand, they avoided going into detail since they did not want to lie to the patient - lying was presumed to place a substantial burden on the doctor-patient relationship. On the other hand, they did not want to reduce the placebo effect by telling the patient too much or using the word "placebo" itself. One physician stated that he preferred to give placebos and lie to the patient than to give medication that would work equally well, but have adverse effects.

*'I don't like lying to people, but sometimes 'the end justifies the means' and sometimes I also tell people that certain medication might have an additional effect if one believes in it, or also the quote 'If it does not hurt* , *it won't do any harm either'. Sometimes it can be seen from this point of view too. But I don't say to the patients, 'Here, this is not real medication, but perhaps if you believe in it, it will help.' If you do that you'll certainly ruin everything. If you declare medication as not being real, there won't be any placebo effect at all.' *(PCP 10, male, 62 years)

### Theme 5: Need for guidelines

Physicians were asked their opinion as to whether they believed guidelines on the use of placebos would be helpful. The ethical dilemma was obvious throughout the interviews. Nevertheless, the interviewed PCPs were divided regarding the question as to whether guidelines should be developed. Some of them did not believe they were warranted since they were still convinced that their methods of treatment were based mainly on pharmaceutical effects and that the placebo effect just provided an unavoidable additional effect. Physicians believe that the presence of guidelines implies a lack of integrity. In contrast, more than half of the interviewed physicians declared that they would welcome wider discussion on the use of placebos in practice.

*'I think recommendations and guidelines aren't bad. We always like guidelines. It is good for us if we know what should be done. That certainly wouldn't be bad.' *(PCP 3, female, 54 years)

*'To my knowledge there's nothing. I think it would make sense if we would discuss the ethical aspects once in a while and define under what circumstances it (placebo use) would be compatible with a medical attitude.' *(PCP 6, male, 60 years) Recommendations were seen as an adequate way to deal with physicians' ambivalent attitude toward the use of placebos. Guidelines or at least consensus papers would ease the perceived ethical dilemma.

## Discussion

### Definition

One of the main findings of our study is that the placebo effect is not always recognised by physicians since their definition of placebo is often restricted to what is formally defined as a pure placebo *('...it is a dummy drug without substance.')*. In placebo literature, a variety of different definitions can be found. For example, one publication defined the placebo as "a sham treatment, such as a pill, liquid, or injection without biological activity, used in pharmacology to control for the activity of a drug. However, in many cases this placebo induces biological or psychological effects in the human" [13]. Another publication defined the placebo intervention as "any therapeutic procedure that has an effect on a patient, symptom, syndrome or disease, but which is objectively without specific activity for the condition being treated" [14]. Both definitions describe a placebo as something that produces placebo effects. In contrast to these wider definitions, the PCPs in our qualitative study defined placebos more narrowly as pharmacologically inactive substances. These definitional differences present a considerable challenge with regard to constructive professional discourse on the role of placebos in clinical practice. It is also important to find a standardised definition of the effect of placebo treatment in order (1) to accurately evaluate the effect of any drug therapy, (2) to compare study results with each other, and finally (3) to draw conclusions about the overall effect of placebo interventions, for example in a systematic review.

### Experiences and awareness

Many physicians reported several specific situations in which they encountered placebo effects *('... I hand him a glass of water, telling him that it contains a substance that will calm him down. Sometimes I have observed that a patient will actually calm down a little.')*. This finding seems to be consistent with results from quantitative surveys examining the frequency of placebo use, although we are aware of the fact that the focus of these surveys was completely different from ours (qualitative vs. quantitative approach). For example, studies from Denmark [6] and Israel [7]reported that more than half of the physicians use placebos. In Denmark, 86% of the PCPs prescribed a placebo at least once within the last year and about half of the physicians on average once a month.

Interestingly, many of the interviewed PCPs initially denied that they have observed a placebo effect. But in the course of the interviews, most of them reported situations in which a placebo effect had occurred. PCPs were not aware that some of the drugs they use are classified as impure placebos.

### Use of Placebos in daily practice

All physicians stated that the response to treatment with a placebo differs according to the nature of the illness and that placebos were mainly used for psychosomatic symptoms.

There is evidence that patients suffering from pain or depression respond particularly well to placebos [15], whereas patients treated for cancer, nervous diseases and substance abuse seem to have a lower than average response [16]. A study of placebo use in lower urinary tract symptoms showed that trials assessing subjective outcomes generally record higher placebo effects than those using objectively measurable outcomes (i.e. positron emission tomography (PET)) [17]. This means that the effect of treatment with a placebo on the patients' subjective feeling of being unwell is greater than the actual objectively measurable healing effect is.

### CAM as placebo treatment

Interestingly, complementary and alternative medicine were mentioned in this context by all PCPs. Most physicians agreed that phytotherapy has both important placebo and pharmaceutical effects. Traditional homeopathy has also been strongly equated with placebo effects *('...there is classical homeopathy, where I think the patient's feeling of being cared for and the relationship to the physician account for much of the effect...')*.

Regarding the different situations in which patients respond to placebo treatments, Kaptchuk et al. [18] concluded in their review that the evidence is too weak to support the existence of 'placebo responders'. Such placebo responders could be patients with a particular disorder who respond well to the administering of a placebo and will also respond to the repeated administering of similar placebos under similar conditions. A placebo responder could also be a person who responds to a placebo in one situation and will also respond in another situation or when using a different type of placebo ritual. The PCPs in our study stated that, according to their observations, they can identify particular characteristics of patients likely to respond to placebo treatment.

Women and patients with psychosomatic disorders were mentioned as likely responders, a finding which has already been reported by Chaput de Saintonge et al. [19] in patients with anxiety disorder.

Interestingly, all primary care physicians emphasised the role of patients' positive expectations, but none mentioned that conditioning is also an important factor, as the current main theory suggests: The placebo effect is perceived on the basis of unconscious conditioning and conscious expectations [13].

### Communication with the patient

Most primary care physicians had a conflict concerning the use of placebos when contrasting the beneficial placebo effect with the aspect that placebos only work when they avoid telling the patient about the placebo procedure *('I don't like lying to people, but sometimes 'the end justifies the means'...')*. Since PCPs were aware of such deception and of thus being in a legal grey zone, they gave the patient only general information in situations in which they used placebos. The PCPs stated that if patients had known that they were being treated with placebos, the placebo would not have been effective. Biller-Andorno suggest a way to use placebos with informed consent by building up a trusting doctor-patient relationship [9]. A recent article summarised the results of open-label placebo treatment [20]. The article concluded that experiments suggest the possibility of clinically significant benefit of prescribing placebos without deception, although questions remain about efficacy. In contrast, a study from Pollo et al. [21] showed that response to expectancies is a major determinant of placebo effects. Both studies indicated that deception might not be necessary to promote a placebo response, but more studies in a practice setting are needed to verify this. In placebo-controlled studies, subjects know that they might be given a placebo, but in clinical practice, patients assume that they are being treated with active medication. One suggestion as to how to inform the patients is "to provide a neurobiological explanation, describing the placebo as a harmless agent, whose administration is meant to bring about improvement in the disorder" [22]. Chaput de Saintonge et al. [19] and Lichtenberg et al. [23] discuss further approaches to using placebo treatment.

### Guidelines

PCPs also expressed ambivalence as to whether the dissemination of ethical guidelines would be helpful. Those who accepted the placebo effect as an important part of medical therapy would appreciate consensus papers or guidelines, whereas some other PCPs stated that the use of placebos should not be regarded as professional behaviour and that guidelines would ignore the individuality of each patient. In the US, for example, the American Medical Association's ruling on placebos provides recommendations on placebo use in clinical practice which "respect patients' autonomy by allowing them to participate actively in the medical decision-making process." [24]. Such recommendations might be an approach to reduce uncertainties in dealing with placebos in daily practice.

### Limitations

Some limitations of this study have to be considered. The aim of qualitative studies is to generate ideas and hypotheses. Due to this methodological approach, quantitative conclusions cannot be drawn. Additionally, it is important to emphasise that the statements in our interviews reflect self-reported behaviour, which may not reflect reality. It is also important to recognise a potential selection bias, because the interviewed PCPs were recruited after having indicated their interest in the topic. Furthermore, our sample is geographically restricted and as such not representative of larger populations. The sample consisted predominantly of rural physicians. However, several studies have shown that there are no differences between physicians from rural and urban areas, and therefore, the location of the practice was assumed not to influence the outcome [8,10,25]. Moreover, the term "placebo" itself, mentioned at the beginning of the interview, may bias physicians and influence the way PCPs describe the role of placebos in their daily practice.

To our knowledge, this is the first study to give a detailed understanding of the use of placebos in primary care using a qualitative analysis of primary care physicians' narratives.

## Conclusions

Many PCPs seem to be unaware that some of the drugs they use are classified as impure placebos. Both effectiveness and legal and ethical aspects seem to interfere substantially with placebo use. Dissemination of guidelines and consensus papers may be an approach but it has to be acknowledged that the topic itself is in conflict with the PCPs' perception of themselves as professional and reliable physicians.

## Competing interests

The authors declare that they have no competing interests.

## Authors' contributions

RF, TR and CH all contributed to the design of the interview guide. RF performed a short pilot study, conducted the interviews, transcribed them and wrote the draft of the manuscript. All authors critically reviewed it and contributed to the final manuscript. All authors have seen and approved the final version of the manuscript.

## Pre-publication history

The pre-publication history for this paper can be accessed here:

http://www.biomedcentral.com/1471-2296/12/11/prepub

## Supplementary Material

Additional file 1 **Interview guide**. The used interview guide translated in English. Click here for file
