# Supplementary material for: The use of pure and impure placebo interventions in primary care - a qualitative approach
Source: BMC Fam Pract. 2011 Mar 24;12:11. doi: 10.1186/1471-2296-12-11 (PMC3068943; doi:10.1186/1471-2296-12-11)
Supplement: Additional file 1 — Interview guide. The used interview guide translated in English. [file 1471-2296-12-11-S1.DOC]

**Additional file 1: Interview guide**

| **Themes** | **Question** |
| --- | --- |
| Definition | 1. What is a placebo? There are different definitions. How would you define this term? |
| Association | 2. Is this term rather positive, neutral or negative for you? |
| Opinions about power of belief | 3. Do you believe that physical complaints can improve only by believing in the effectiveness of a therapy?  (What are „physical complaints“? e.g. pain after an operation, bronchoconstriction in bronchial asthma) |
| Experiences | 4.1. Can you tell from your experience as a primary care physician if a placebo effect exists?  4.2. Do you have experience in homeopathy or phytotherapy? |
| Applications  Placebo therapy examples from practice (pure placebos)  Examples of placebo therapies from the practice (partial placebos) | 5.1 Do you have placebos such as sugar pills stocked in your practice?  5.2 Does it occur that you deliberately use procedures or pharmacologically effective therapies (medications) in order to exploit placebo effects? |
| Estimation of effect | 6. Do you think that the placebo effect is of different intensity in various diseases? |
| Moral aspect „lying“ | 7. It is to be presumed that the effect of a placebo is less or not at all present if you openly tell the patients that it's a placebo. To achieve a maximal effect, you have to – strictly speaking – tell the patient a fib. How do you feel about that? |
| Example | 8. There are prescription drugs whose benefit is doubted by scientific studies (e.g. vitamin supplements without demonstrated vitamin deficiency, yeast extracts in bowel disturbances, mistletoe extracts in malignancies).  In case a patient requests a drug from you which you deem questionable in his case – when do you comply with his request and when don't you? |
| Future perspectives | 9. Do you think that this topic of placebos should be more exhaustively discussed in the medical profession, and that perhaps even recommendations or guidelines should be developed? |
